# 2,2-Bis[3,5-bis­(di­methyl­amino)­phen­yl]-1,1,1,3,3,3-hexa­methyl­tris­ilane

**DOI:** 10.1107/S2414314620012997

**Published:** 2020-09-25

**Authors:** Yoshiyuki Mizuhata, Kento Iwai, Norihiro Tokitoh

**Affiliations:** a Institute for Chemical Research, Kyoto University, Gokasho, Uji, Kyoto 611-0011, Japan; Okayama University, Japan

**Keywords:** crystal structure, tris­ilane, 3,5-bis(di­methyl­amino)­phen­yl

## Abstract

The title compound, C_26_H_48_N_4_Si_3_, was synthesized by the reaction of 2,2-di­chloro­tris­ilane with 3,5-bis­(di­methyl­amino)­phenyl­lithium. In the mol­ecule, the dihedral angle between the benzene rings is 57.57 (7)° and the Si—Si—Si bond angle is 110.08 (2)°. In the crystal, mol­ecules are linked *via* an SiC–H⋯π(ar­yl) inter­action, forming a chain along the *c-*axis direction.

## Abstract 2

The mol­ecules of the title compound, C_26_H_48_N_4_Si_3_, are linked *via* an SiC–H⋯π(ar­yl) inter­action in the crystal.

## Graphical abstract

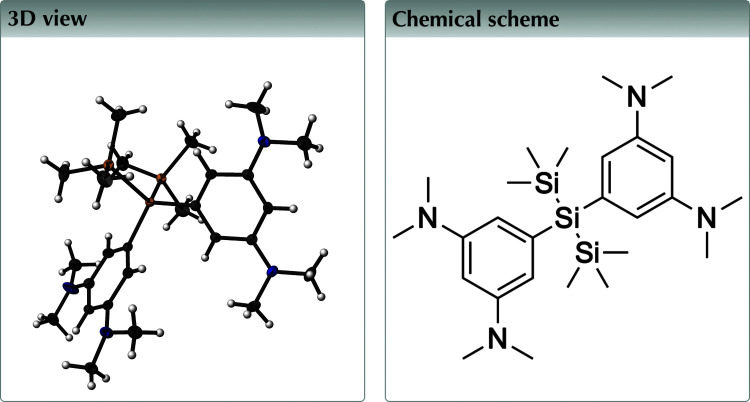

## Structure description

Trisilane derivatives are often used as a precursor for divalent silicon compounds (silylenes) (Gaspar & West, 1998 ▸). Photoirradiation of a tris­ilane results in the elimination of a disilane derived from the silyl moieties of the both ends and the generation of a silylene from the central one. Silylenes are highly reactive and known to give the dimer, silicon–silicon double–bond compound (disilene) (West *et al.*, 1981 ▸), or higher oligomers without trapping reagent. Herein, we describe the synthesis and structural characterization of a novel tris­ilane bearing two 3,5-bis­(di­methyl­amino)­phenyl substituents on the central silicon atom.

**Figure 1:**
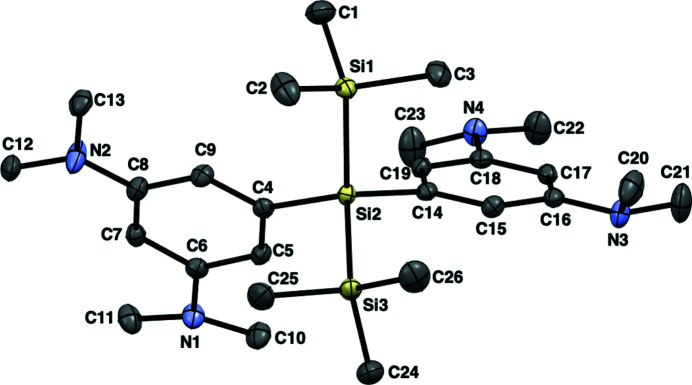
The mol­ecular structure of the title compound, showing displacement ellipsoids at the 50% probability level. Hydrogen atoms are omitted for clarity.

**Figure 2:**
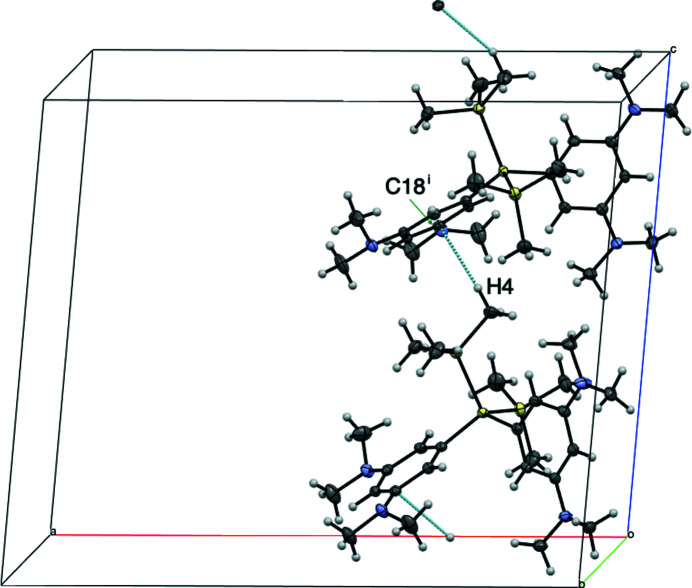
A packing diagram of the title compound, emphasizing the inter­molecular SiC—H⋯π(ar­yl) inter­actions (light-blue dotted lines). [Symmetry code: (i) *x*, −*y* + 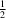 , *z* + 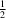 .]

In the mol­ecule (Fig. 1 ▸), the Si—Si bond lengths are 2.3488 (5) and 2.3465 (5) Å, which are close to the shortest bond lengths among those of the reported 2,2-diaryl-1,1,1,3,3,3-hexa­methyl­tris­ilanes (2.347–2.397 Å; Archibald *et al.*, 1993 ▸; Lange *et al.*, 1991 ▸; Millevolte *et al.*, 1997 ▸; Pusztai *et al.*, 2013 ▸). The sums of the angles around the nitro­gen atoms (N1, N2, N3 and N4) are 353.8, 358.9, 344.6 and 350.5°, respectively, indicating that the geometries around the nitro­gen atoms on one aryl substituent with N3/N4 are more pyramidalized than those on the other with N1/N2. In the crystal, mol­ecules are linked by an SiC–H⋯π(ar­yl) inter­action (Fig. 2 ▸ and Table 1 ▸).

## Synthesis and crystallization

To a solution of 1-bromo-3,5-bis­(di­methyl­amino)­benzene (194.6 mg, 0.80 mmol) in Et_2_O (6.0 ml) was added *t*-BuLi (1.64 *M* in pentane, 1.07 ml, 1.76 mmol) at −78 °C, and the solution was stirred for 1 h at the same temperature. Then, 2,2-di­chloro-1,1,1,3,3,3-hexa­methyl­tris­ilane (104.2 mg, 0.42 mmol) in Et_2_O (4 ml) was added dropwise, and the reaction mixture was gradually warmed up to room temperature over 14 h. After quenching with a saturated aqueous solution of NH_4_Cl (10 ml), the water phase was extracted with Et_2_O (10 ml × 2). The combined organic phase was dried over Na_2_SO_4_ and evaporated *in vacuo*. Finally, recrystallization from the mixed solvents of hexa­ne/di­chloro­methane gave the title compound (101.3 mg, 0.21 mmol, 50%) as colorless crystals. ^1^H NMR (300 Hz, CDCl_3_) *δ* 6.40 (*d*, *J* = 1.2 Hz, 4H), 6.11 (*t*, *J* = 1.2 Hz, 2H), 2.90 (*s*, 24H), 0.19 (*s*, 18H); ^13^C NMR (75.5 Hz, CDCl_3_) *δ* 151.1 (CH), 136.7 (C), 110.9 (CH), 98.6 (C), 41.1 (NCH_3_), −0.012 (SiCH_3_).

## Refinement

Crystal data, data collection and structure refinement details are summarized in Table 2 ▸.

## Supplementary Material

Crystal structure: contains datablock(s) I. DOI: 10.1107/S2414314620012997/is4044sup1.cif

Structure factors: contains datablock(s) I. DOI: 10.1107/S2414314620012997/is4044Isup2.hkl

Supporting information file. DOI: 10.1107/S2414314620012997/is4044Isup3.cml

CCDC reference: 2033547

Additional supporting information:  crystallographic information; 3D view; checkCIF report

## Figures and Tables

**Table 1:** Hydrogen-bond geometry (Å, °) *Cg* is the centroid of the C14–C19 ring.

*D*—H⋯*A*	*D*—H	H⋯*A*	*D*⋯*A*	*D*—H⋯*A*
C2—H4⋯*Cg* ^i^	0.98	2.81	3.7869 (17)	172

Symmetry code: (i) 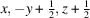 .

**Table 2:** Experimental details

Crystal data	
Chemical formula	C_26_H_48_N_4_Si_3_
*M*_r_	500.95
Crystal system, space group	Monoclinic, *P*2_1_/*c*
Temperature (K)	103
*a*, *b*, *c* (Å)	20.6543 (3), 8.3650 (1), 17.5215 (3)
β (°)	97.371 (2)
*V* (Å^3^)	3002.23 (8)
*Z*	4
Radiation type	Mo *K*α
μ (mm^−1^)	0.18
Crystal size (mm)	0.20 × 0.20 × 0.20

Data collection	
Diffractometer	Rigaku Saturn
Absorption correction	Multi-scan (*CrysAlis PRO* (Rigaku OD, 2018 ▸)
*T*_min_, *T*_max_	0.977, 1.000
No. of measured, independent and observed [*I* > 2σ(*I*)] reflections	33409, 5581, 4877
*R*_int_	0.029
(sin θ/λ)_max_ (Å^−1^)	0.606

Refinement	
*R*[*F* ^2^ > 2σ(*F* ^2^)], *wR*(*F* ^2^), *S*	0.033, 0.086, 1.04
No. of reflections	5581
No. of parameters	312
H-atom treatment	H-atom parameters constrained
Δρ_max_, Δρ_min_ (e Å^−3^)	0.38, −0.18

Computer programs: *CrystalClear* (Rigaku, 1999 ▸), *CrysAlis PRO* (Rigaku OD, 2018 ▸), *SHELXT* (Sheldrick, 2015*a* ▸), *SHELXL2018/1* (Sheldrick, 2015*b* ▸), *Yadokari-XG* (Kabuto *et al.*, 2009 ▸) and *Mercury* (Macrae *et al.*, 2020 ▸).

## full crystallographic data

### Crystal data

**Table:**

C_26_H_48_N_4_Si_3_	*F*(000) = 1096
*M**_r_* = 500.95	*D*_x_ = 1.108 Mg m^−^^3^
Monoclinic, *P*2_1_/*c*	Mo *K*α radiation, λ = 0.71073 Å
*a* = 20.6543 (3) Å	Cell parameters from 24488 reflections
*b* = 8.3650 (1) Å	θ = 1.8–30.3°
*c* = 17.5215 (3) Å	µ = 0.18 mm^−^^1^
β = 97.371 (2)°	*T* = 103 K
*V* = 3002.23 (8) Å^3^	Prism, colorless
*Z* = 4	0.20 × 0.20 × 0.20 mm

### Data collection

**Table:**

Rigaku Saturn diffractometer	5581 independent reflections
Radiation source: fine-focus sealed X-ray tube	4877 reflections with *I* > 2σ(*I*)
Graphite monochromator	*R*_int_ = 0.029
Detector resolution: 28.5714 pixels mm^-1^	θ_max_ = 25.5°, θ_min_ = 2.0°
ω scans	*h* = −25→25
Absorption correction: multi-scan (CrysAlisPro (Rigaku OD, 2018)	*k* = −10→10
*T*_min_ = 0.977, *T*_max_ = 1.000	*l* = −21→21
33409 measured reflections	

### Refinement

**Table:**

Refinement on *F*^2^	Primary atom site location: dual
Least-squares matrix: full	Secondary atom site location: difference Fourier map
*R*[*F*^2^ > 2σ(*F*^2^)] = 0.033	Hydrogen site location: inferred from neighbouring sites
*wR*(*F*^2^) = 0.086	H-atom parameters constrained
*S* = 1.04	*w* = 1/[σ^2^(*F*_o_^2^) + (0.0427*P*)^2^ + 1.4907*P*] where *P* = (*F*_o_^2^ + 2*F*_c_^2^)/3
5581 reflections	(Δ/σ)_max_ = 0.001
312 parameters	Δρ_max_ = 0.38 e Å^−^^3^
0 restraints	Δρ_min_ = −0.18 e Å^−^^3^

### Special details

**Table:**

Geometry. All esds (except the esd in the dihedral angle between two l.s. planes) are estimated using the full covariance matrix. The cell esds are taken into account individually in the estimation of esds in distances, angles and torsion angles; correlations between esds in cell parameters are only used when they are defined by crystal symmetry. An approximate (isotropic) treatment of cell esds is used for estimating esds involving l.s. planes.

### Fractional atomic coordinates and isotropic or equivalent isotropic displacement parameters (Å^2^)

**Table:**

	*x*	*y*	*z*	*U*_iso_*/*U*_eq_	
Si1	0.30425 (2)	0.26359 (5)	0.40578 (2)	0.01776 (11)	
Si2	0.25081 (2)	0.23923 (4)	0.27966 (2)	0.01378 (10)	
Si3	0.20713 (2)	−0.01864 (5)	0.26001 (2)	0.01934 (11)	
C1	0.32187 (9)	0.4774 (2)	0.43281 (10)	0.0326 (4)	
H1	0.342782	0.529781	0.392332	0.049*	
H2	0.351105	0.481717	0.481460	0.049*	
H3	0.280943	0.532430	0.438785	0.049*	
C2	0.25745 (8)	0.1727 (2)	0.47983 (9)	0.0338 (4)	
H4	0.282593	0.183946	0.530913	0.051*	
H5	0.249861	0.059114	0.468290	0.051*	
H6	0.215449	0.227615	0.478973	0.051*	
C3	0.38414 (7)	0.1570 (2)	0.40627 (9)	0.0278 (4)	
H7	0.409142	0.164627	0.457597	0.042*	
H8	0.408976	0.206399	0.368441	0.042*	
H9	0.376058	0.044327	0.393031	0.042*	
C4	0.17831 (7)	0.37787 (16)	0.26124 (8)	0.0157 (3)	
C5	0.15255 (7)	0.42232 (16)	0.18664 (8)	0.0166 (3)	
H10	0.174097	0.391031	0.144264	0.020*	
C6	0.09489 (7)	0.51314 (16)	0.17395 (8)	0.0164 (3)	
C7	0.06416 (7)	0.55949 (17)	0.23737 (8)	0.0175 (3)	
H11	0.025674	0.622675	0.229263	0.021*	
C8	0.08913 (7)	0.51440 (17)	0.31244 (8)	0.0189 (3)	
C9	0.14687 (7)	0.42416 (17)	0.32341 (8)	0.0181 (3)	
H12	0.164780	0.394190	0.374040	0.022*	
N1	0.06746 (6)	0.55413 (15)	0.09955 (7)	0.0211 (3)	
C10	0.10803 (8)	0.5412 (2)	0.03772 (8)	0.0247 (3)	
H13	0.120610	0.429339	0.031923	0.037*	
H14	0.147335	0.606661	0.050088	0.037*	
H15	0.083461	0.578969	−0.010478	0.037*	
C11	0.01575 (7)	0.6719 (2)	0.08958 (9)	0.0252 (3)	
H16	0.032046	0.773793	0.112060	0.038*	
H17	−0.020940	0.635214	0.115365	0.038*	
H18	0.001115	0.686560	0.034579	0.038*	
N2	0.05745 (7)	0.55862 (18)	0.37463 (7)	0.0305 (3)	
C12	0.00044 (7)	0.65851 (19)	0.36342 (9)	0.0235 (3)	
H19	−0.032513	0.609127	0.325453	0.035*	
H20	0.012242	0.763704	0.344817	0.035*	
H21	−0.017287	0.670837	0.412357	0.035*	
C13	0.08889 (8)	0.5299 (2)	0.45176 (8)	0.0253 (3)	
H22	0.131207	0.584552	0.459169	0.038*	
H23	0.095511	0.414821	0.459639	0.038*	
H24	0.061305	0.570997	0.488874	0.038*	
C14	0.31425 (7)	0.28489 (17)	0.21410 (8)	0.0151 (3)	
C15	0.35872 (7)	0.16651 (17)	0.19910 (8)	0.0170 (3)	
H25	0.353827	0.060958	0.217532	0.020*	
C16	0.41035 (7)	0.20138 (17)	0.15730 (8)	0.0169 (3)	
C17	0.41619 (7)	0.35685 (17)	0.12925 (8)	0.0167 (3)	
H26	0.450754	0.381126	0.100236	0.020*	
C18	0.37191 (7)	0.47750 (17)	0.14323 (8)	0.0162 (3)	
C19	0.32122 (7)	0.43937 (17)	0.18636 (8)	0.0160 (3)	
H27	0.291203	0.520064	0.196795	0.019*	
N3	0.45454 (6)	0.07908 (15)	0.14290 (7)	0.0215 (3)	
N4	0.37992 (6)	0.63483 (15)	0.11844 (7)	0.0219 (3)	
C20	0.47693 (9)	−0.0230 (2)	0.20856 (10)	0.0358 (4)	
H28	0.439219	−0.073086	0.227626	0.054*	
H29	0.500814	0.041608	0.249542	0.054*	
H30	0.505801	−0.106202	0.192602	0.054*	
C21	0.50722 (8)	0.1230 (2)	0.09949 (11)	0.0366 (4)	
H31	0.536208	0.199436	0.129305	0.055*	
H32	0.488974	0.171889	0.050608	0.055*	
H33	0.532024	0.027143	0.089272	0.055*	
C22	0.43019 (8)	0.6685 (2)	0.07004 (10)	0.0313 (4)	
H34	0.419445	0.614702	0.020332	0.047*	
H35	0.472326	0.629362	0.095198	0.047*	
H36	0.432763	0.784068	0.061867	0.047*	
C23	0.32265 (9)	0.7339 (2)	0.10043 (12)	0.0361 (4)	
H37	0.294343	0.722489	0.140908	0.054*	
H38	0.298835	0.700536	0.051007	0.054*	
H39	0.335941	0.845920	0.097152	0.054*	
C24	0.18019 (8)	−0.0508 (2)	0.15471 (10)	0.0310 (4)	
H40	0.146709	0.028127	0.136380	0.047*	
H41	0.162104	−0.158697	0.146642	0.047*	
H42	0.217687	−0.038664	0.126137	0.047*	
C25	0.13399 (8)	−0.0289 (2)	0.31312 (10)	0.0332 (4)	
H43	0.148027	−0.019567	0.368531	0.050*	
H44	0.111634	−0.131258	0.302292	0.050*	
H45	0.104091	0.058891	0.296305	0.050*	
C26	0.26437 (9)	−0.18132 (19)	0.29957 (11)	0.0340 (4)	
H46	0.242957	−0.285333	0.290345	0.051*	
H47	0.276191	−0.165481	0.355026	0.051*	
H48	0.303862	−0.178120	0.274009	0.051*	

### Atomic displacement parameters (Å^2^)

**Table:**

	*U*^11^	*U*^22^	*U*^33^	*U*^12^	*U*^13^	*U*^23^
Si1	0.0165 (2)	0.0216 (2)	0.0150 (2)	−0.00027 (16)	0.00150 (15)	0.00142 (15)
Si2	0.01451 (19)	0.01279 (19)	0.0143 (2)	0.00137 (14)	0.00278 (15)	0.00109 (14)
Si3	0.0186 (2)	0.0153 (2)	0.0246 (2)	−0.00180 (15)	0.00459 (16)	−0.00247 (16)
C1	0.0323 (9)	0.0292 (9)	0.0344 (9)	−0.0007 (7)	−0.0026 (7)	−0.0108 (7)
C2	0.0262 (9)	0.0523 (11)	0.0230 (8)	0.0001 (8)	0.0038 (7)	0.0145 (8)
C3	0.0216 (8)	0.0364 (9)	0.0241 (8)	0.0064 (7)	−0.0022 (6)	0.0008 (7)
C4	0.0161 (7)	0.0121 (7)	0.0191 (7)	−0.0007 (5)	0.0024 (5)	0.0003 (5)
C5	0.0185 (7)	0.0161 (7)	0.0161 (7)	0.0002 (6)	0.0055 (5)	−0.0011 (5)
C6	0.0174 (7)	0.0141 (7)	0.0174 (7)	−0.0013 (5)	0.0017 (5)	0.0016 (5)
C7	0.0161 (7)	0.0160 (7)	0.0205 (7)	0.0026 (5)	0.0031 (6)	0.0010 (6)
C8	0.0213 (7)	0.0179 (7)	0.0184 (7)	0.0018 (6)	0.0058 (6)	−0.0005 (6)
C9	0.0215 (7)	0.0187 (7)	0.0142 (7)	0.0025 (6)	0.0022 (6)	0.0020 (6)
N1	0.0211 (6)	0.0263 (7)	0.0160 (6)	0.0063 (5)	0.0023 (5)	0.0031 (5)
C10	0.0269 (8)	0.0313 (9)	0.0160 (7)	0.0049 (7)	0.0036 (6)	0.0021 (6)
C11	0.0208 (8)	0.0324 (9)	0.0221 (8)	0.0058 (7)	0.0017 (6)	0.0082 (7)
N2	0.0318 (8)	0.0440 (9)	0.0167 (7)	0.0209 (7)	0.0063 (6)	0.0012 (6)
C12	0.0197 (7)	0.0287 (8)	0.0234 (8)	0.0037 (6)	0.0076 (6)	−0.0035 (6)
C13	0.0286 (8)	0.0308 (9)	0.0174 (7)	0.0046 (7)	0.0065 (6)	−0.0015 (6)
C14	0.0162 (7)	0.0170 (7)	0.0120 (6)	−0.0003 (5)	0.0009 (5)	−0.0009 (5)
C15	0.0205 (7)	0.0139 (7)	0.0168 (7)	0.0003 (6)	0.0029 (6)	0.0012 (5)
C16	0.0165 (7)	0.0186 (7)	0.0151 (7)	0.0019 (6)	−0.0001 (5)	−0.0027 (6)
C17	0.0147 (7)	0.0210 (7)	0.0145 (7)	−0.0017 (6)	0.0026 (5)	−0.0009 (6)
C18	0.0179 (7)	0.0160 (7)	0.0143 (7)	−0.0020 (6)	0.0000 (5)	−0.0009 (5)
C19	0.0167 (7)	0.0150 (7)	0.0162 (7)	0.0017 (5)	0.0020 (5)	−0.0013 (5)
N3	0.0209 (6)	0.0216 (6)	0.0235 (7)	0.0063 (5)	0.0081 (5)	0.0010 (5)
N4	0.0241 (7)	0.0167 (6)	0.0263 (7)	−0.0010 (5)	0.0091 (5)	0.0041 (5)
C20	0.0400 (10)	0.0374 (10)	0.0298 (9)	0.0231 (8)	0.0042 (7)	0.0039 (8)
C21	0.0317 (9)	0.0311 (9)	0.0515 (11)	0.0105 (8)	0.0228 (8)	0.0035 (8)
C22	0.0336 (9)	0.0212 (8)	0.0424 (10)	−0.0047 (7)	0.0176 (8)	0.0052 (7)
C23	0.0356 (10)	0.0219 (8)	0.0543 (12)	0.0070 (7)	0.0190 (9)	0.0157 (8)
C24	0.0274 (9)	0.0335 (9)	0.0315 (9)	0.0003 (7)	0.0012 (7)	−0.0130 (7)
C25	0.0278 (9)	0.0335 (9)	0.0405 (10)	−0.0119 (7)	0.0129 (7)	−0.0069 (8)
C26	0.0376 (10)	0.0169 (8)	0.0474 (11)	0.0013 (7)	0.0056 (8)	0.0039 (7)

### Geometric parameters (Å, º)

**Table:**

Si1—C1	1.8737 (17)	C12—H20	0.9800
Si1—C3	1.8744 (16)	C12—H21	0.9800
Si1—C2	1.8755 (16)	C13—H22	0.9800
Si1—Si2	2.3488 (5)	C13—H23	0.9800
Si2—C4	1.8887 (14)	C13—H24	0.9800
Si2—C14	1.8890 (14)	C14—C19	1.395 (2)
Si2—Si3	2.3465 (5)	C14—C15	1.398 (2)
Si3—C25	1.8751 (17)	C15—C16	1.400 (2)
Si3—C26	1.8762 (17)	C15—H25	0.9500
Si3—C24	1.8767 (17)	C16—C17	1.401 (2)
C1—H1	0.9800	C16—N3	1.4151 (18)
C1—H2	0.9800	C17—C18	1.404 (2)
C1—H3	0.9800	C17—H26	0.9500
C2—H4	0.9800	C18—N4	1.4023 (18)
C2—H5	0.9800	C18—C19	1.404 (2)
C2—H6	0.9800	C19—H27	0.9500
C3—H7	0.9800	N3—C21	1.452 (2)
C3—H8	0.9800	N3—C20	1.460 (2)
C3—H9	0.9800	N4—C23	1.446 (2)
C4—C9	1.393 (2)	N4—C22	1.4499 (19)
C4—C5	1.3971 (19)	C20—H28	0.9800
C5—C6	1.406 (2)	C20—H29	0.9800
C5—H10	0.9500	C20—H30	0.9800
C6—N1	1.3959 (18)	C21—H31	0.9800
C6—C7	1.403 (2)	C21—H32	0.9800
C7—C8	1.402 (2)	C21—H33	0.9800
C7—H11	0.9500	C22—H34	0.9800
C8—N2	1.3911 (19)	C22—H35	0.9800
C8—C9	1.404 (2)	C22—H36	0.9800
C9—H12	0.9500	C23—H37	0.9800
N1—C11	1.4473 (19)	C23—H38	0.9800
N1—C10	1.4562 (19)	C23—H39	0.9800
C10—H13	0.9800	C24—H40	0.9800
C10—H14	0.9800	C24—H41	0.9800
C10—H15	0.9800	C24—H42	0.9800
C11—H16	0.9800	C25—H43	0.9800
C11—H17	0.9800	C25—H44	0.9800
C11—H18	0.9800	C25—H45	0.9800
N2—C12	1.4365 (19)	C26—H46	0.9800
N2—C13	1.4423 (19)	C26—H47	0.9800
C12—H19	0.9800	C26—H48	0.9800
			
C1—Si1—C3	108.08 (8)	N2—C12—H21	109.5
C1—Si1—C2	108.21 (9)	H19—C12—H21	109.5
C3—Si1—C2	109.61 (8)	H20—C12—H21	109.5
C1—Si1—Si2	111.92 (6)	N2—C13—H22	109.5
C3—Si1—Si2	105.68 (5)	N2—C13—H23	109.5
C2—Si1—Si2	113.20 (6)	H22—C13—H23	109.5
C4—Si2—C14	111.62 (6)	N2—C13—H24	109.5
C4—Si2—Si3	104.95 (4)	H22—C13—H24	109.5
C14—Si2—Si3	112.35 (5)	H23—C13—H24	109.5
C4—Si2—Si1	111.97 (5)	C19—C14—C15	119.40 (13)
C14—Si2—Si1	106.00 (4)	C19—C14—Si2	120.66 (10)
Si3—Si2—Si1	110.08 (2)	C15—C14—Si2	119.65 (10)
C25—Si3—C26	107.05 (8)	C14—C15—C16	120.91 (13)
C25—Si3—C24	108.98 (8)	C14—C15—H25	119.5
C26—Si3—C24	110.64 (8)	C16—C15—H25	119.5
C25—Si3—Si2	106.75 (6)	C15—C16—C17	118.86 (13)
C26—Si3—Si2	113.60 (6)	C15—C16—N3	119.66 (13)
C24—Si3—Si2	109.63 (6)	C17—C16—N3	121.47 (13)
Si1—C1—H1	109.5	C16—C17—C18	121.24 (13)
Si1—C1—H2	109.5	C16—C17—H26	119.4
H1—C1—H2	109.5	C18—C17—H26	119.4
Si1—C1—H3	109.5	N4—C18—C19	120.37 (13)
H1—C1—H3	109.5	N4—C18—C17	121.00 (13)
H2—C1—H3	109.5	C19—C18—C17	118.54 (13)
Si1—C2—H4	109.5	C14—C19—C18	121.03 (13)
Si1—C2—H5	109.5	C14—C19—H27	119.5
H4—C2—H5	109.5	C18—C19—H27	119.5
Si1—C2—H6	109.5	C16—N3—C21	117.02 (12)
H4—C2—H6	109.5	C16—N3—C20	115.40 (12)
H5—C2—H6	109.5	C21—N3—C20	112.20 (13)
Si1—C3—H7	109.5	C18—N4—C23	118.68 (12)
Si1—C3—H8	109.5	C18—N4—C22	119.02 (12)
H7—C3—H8	109.5	C23—N4—C22	112.84 (13)
Si1—C3—H9	109.5	N3—C20—H28	109.5
H7—C3—H9	109.5	N3—C20—H29	109.5
H8—C3—H9	109.5	H28—C20—H29	109.5
C9—C4—C5	119.86 (13)	N3—C20—H30	109.5
C9—C4—Si2	118.43 (10)	H28—C20—H30	109.5
C5—C4—Si2	121.45 (11)	H29—C20—H30	109.5
C4—C5—C6	120.33 (13)	N3—C21—H31	109.5
C4—C5—H10	119.8	N3—C21—H32	109.5
C6—C5—H10	119.8	H31—C21—H32	109.5
N1—C6—C7	120.08 (13)	N3—C21—H33	109.5
N1—C6—C5	120.96 (13)	H31—C21—H33	109.5
C7—C6—C5	118.95 (13)	H32—C21—H33	109.5
C8—C7—C6	121.30 (13)	N4—C22—H34	109.5
C8—C7—H11	119.4	N4—C22—H35	109.5
C6—C7—H11	119.4	H34—C22—H35	109.5
N2—C8—C7	120.67 (13)	N4—C22—H36	109.5
N2—C8—C9	120.83 (13)	H34—C22—H36	109.5
C7—C8—C9	118.50 (13)	H35—C22—H36	109.5
C4—C9—C8	121.05 (13)	N4—C23—H37	109.5
C4—C9—H12	119.5	N4—C23—H38	109.5
C8—C9—H12	119.5	H37—C23—H38	109.5
C6—N1—C11	118.99 (12)	N4—C23—H39	109.5
C6—N1—C10	118.25 (12)	H37—C23—H39	109.5
C11—N1—C10	116.55 (12)	H38—C23—H39	109.5
N1—C10—H13	109.5	Si3—C24—H40	109.5
N1—C10—H14	109.5	Si3—C24—H41	109.5
H13—C10—H14	109.5	H40—C24—H41	109.5
N1—C10—H15	109.5	Si3—C24—H42	109.5
H13—C10—H15	109.5	H40—C24—H42	109.5
H14—C10—H15	109.5	H41—C24—H42	109.5
N1—C11—H16	109.5	Si3—C25—H43	109.5
N1—C11—H17	109.5	Si3—C25—H44	109.5
H16—C11—H17	109.5	H43—C25—H44	109.5
N1—C11—H18	109.5	Si3—C25—H45	109.5
H16—C11—H18	109.5	H43—C25—H45	109.5
H17—C11—H18	109.5	H44—C25—H45	109.5
C8—N2—C12	120.48 (12)	Si3—C26—H46	109.5
C8—N2—C13	119.29 (12)	Si3—C26—H47	109.5
C12—N2—C13	119.17 (12)	H46—C26—H47	109.5
N2—C12—H19	109.5	Si3—C26—H48	109.5
N2—C12—H20	109.5	H46—C26—H48	109.5
H19—C12—H20	109.5	H47—C26—H48	109.5
			
C14—Si2—C4—C9	−145.45 (11)	C4—Si2—C14—C19	28.69 (13)
Si3—Si2—C4—C9	92.60 (11)	Si3—Si2—C14—C19	146.27 (10)
Si1—Si2—C4—C9	−26.80 (12)	Si1—Si2—C14—C19	−93.46 (11)
C14—Si2—C4—C5	40.35 (13)	C4—Si2—C14—C15	−157.41 (11)
Si3—Si2—C4—C5	−81.60 (12)	Si3—Si2—C14—C15	−39.83 (12)
Si1—Si2—C4—C5	159.00 (10)	Si1—Si2—C14—C15	80.44 (11)
C9—C4—C5—C6	−0.2 (2)	C19—C14—C15—C16	0.6 (2)
Si2—C4—C5—C6	173.90 (10)	Si2—C14—C15—C16	−173.39 (10)
C4—C5—C6—N1	−177.96 (13)	C14—C15—C16—C17	−1.2 (2)
C4—C5—C6—C7	0.6 (2)	C14—C15—C16—N3	−179.91 (12)
N1—C6—C7—C8	177.34 (13)	C15—C16—C17—C18	0.8 (2)
C5—C6—C7—C8	−1.3 (2)	N3—C16—C17—C18	179.53 (12)
C6—C7—C8—N2	−178.75 (14)	C16—C17—C18—N4	176.69 (13)
C6—C7—C8—C9	1.4 (2)	C16—C17—C18—C19	0.1 (2)
C5—C4—C9—C8	0.4 (2)	C15—C14—C19—C18	0.4 (2)
Si2—C4—C9—C8	−173.87 (11)	Si2—C14—C19—C18	174.32 (10)
N2—C8—C9—C4	179.17 (14)	N4—C18—C19—C14	−177.34 (13)
C7—C8—C9—C4	−1.0 (2)	C17—C18—C19—C14	−0.7 (2)
C7—C6—N1—C11	13.1 (2)	C15—C16—N3—C21	−179.75 (14)
C5—C6—N1—C11	−168.37 (13)	C17—C16—N3—C21	1.6 (2)
C7—C6—N1—C10	164.45 (13)	C15—C16—N3—C20	−44.38 (19)
C5—C6—N1—C10	−17.0 (2)	C17—C16—N3—C20	136.94 (15)
C7—C8—N2—C12	−3.3 (2)	C19—C18—N4—C23	−32.2 (2)
C9—C8—N2—C12	176.48 (14)	C17—C18—N4—C23	151.30 (15)
C7—C8—N2—C13	−171.42 (14)	C19—C18—N4—C22	−176.24 (13)
C9—C8—N2—C13	8.4 (2)	C17—C18—N4—C22	7.3 (2)

### Hydrogen-bond geometry (Å, º)

*Cg* is the centroid of the C14–C19 ring.

**Table:**

*D*—H···*A*	*D*—H	H···*A*	*D*···*A*	*D*—H···*A*
C2—H4···*Cg*^i^	0.98	2.81	3.7869 (17)	172

Symmetry code: (i) *x*, −*y*+1/2, *z*+1/2.
